# Molecular Detection of Filarioid Nematodes in Bats from the Western Brazilian Amazon

**DOI:** 10.1007/s11686-026-01398-8

**Published:** 2026-09-07

**Authors:** Jhonatan Henrique Lima da Rocha, Pedro Zanata Lima dos Santos, Cleyton Silva de Araújo, Guilherme Henrique Reckziegel, Deise Kelly Alves da Silva, Laura Kelly Teixeira Veras, Samyly Coutinho de Aguiar Silva, Sérgio Luiz Bessa Luz, Tamyres Izarelly Barbosa da Silva

**Affiliations:** 1 Laboratório de Doenças Infecciosas dos Animais (LADIA), Universidade Federal do Acre (UFAC), Rodovia, BR-364, Km 04, Distrito Industrial, Rio Branco, 69920-900 AC Brazil; 2 Núcleo de Patógenos, Reservatórios e Vetores na Amazônia – PReV Amazônia, Fiocruz Amazônia, Manaus, AM Brazil; 3 Programa de Pós-Graduação em Biologia Parasitária, Instituto Oswaldo Cruz (IOC), Fiocruz, Rio de Janeiro, RJ Brazil; 4 Center for Biological and Natural Sciences (CCBN), Universidade Federal do Acre (UFAC), Rio Branco, AC Brazil; 5 Programa de Pós-Graduação em Biologia da Interação Patógeno- Hospedeiro (PPGBIO Interação), Fiocruz Amazônia, Manaus, AM Brazil; 6 Fundação de Vigilância em Saúde do Amazonas – Drª Rosemary Costa Pinto (FVS-RCP), Manaus, AM Brazil

**Keywords:** Amazonia, Chiroptera, Filarioidea, Onchocercidae, Parasite diversity

## Abstract

**Purpose:**

Filarial nematodes (Onchocercidae) infect diverse mammals, but their occurrence and molecular diversity in Amazonian bats remain poorly documented. We investigated bat-associated filarioids in urban and peri-urban forest fragments in Rio Branco, Acre, western Brazilian Amazon.

**Methods:**

Between December 2022 and October 2023, bats were captured with mist nets in five forest fragments and identified morphologically. Blood was screened for microfilariae by thin smear and microhematocrit. A mitochondrial 12 S rRNA fragment (~ 330 bp) was amplified, and PCR-positive products were Sanger sequenced. Consensus sequences were assessed by BLASTn; after alignment and trimming, 300 nucleotide positions were retained for pairwise p-distance analysis and maximum-likelihood phylogenetic inference (HKY + G; 1,000 bootstraps).

**Results:**

Ninety bats representing 20 species, 13 genera, and four families were captured. Microfilariae were detected in 2/90 bats (2.2%): *Artibeus planirostris* and *Tonatia maresi*. UDV06 (PZ124269), from *A. planirostris*, showed strong affinity with *Litomosoides*, with ~ 98–99% BLASTn identity, complete query coverage, low divergence, and clustering within the genus. HF17 (PZ124268), from *T. maresi*, showed only ~ 87–88% identity to available *Litomosoides* sequences and a markedly divergent phylogenetic position, supporting its conservative treatment as an unresolved member of Onchocercidae.

**Conclusion:**

These findings document bat-associated filarioid occurrence and molecular diversity in the western Brazilian Amazon. UDV06 is supported as *Litomosoides* at the genus level, whereas HF17 remains unresolved within Onchocercidae. Additional mitochondrial, particularly *cox1*, and nuclear markers are needed for improved taxonomic resolution.

## Introduction

Filarioid nematodes (Nematoda: Filarioidea), particularly members of the family Onchocercidae, include lineages of major medical and veterinary importance as well as a diverse assemblage of wildlife-associated taxa that remain poorly characterized in tropical ecosystems [1]. Environmental change in the Amazon can alter host–vector contact patterns and infectious-disease dynamics [2, 3], reinforcing the value of baseline parasitological surveys in wildlife.

Brazil harbors one of the richest bat faunas in the world, with 186 species currently recognized across nine families [4]. Within the Brazilian Amazon, a recent synthesis of research conducted over the past decade confirmed records of 144 bat species, distributed across 63 genera and nine families, and underscored that current knowledge remains geographically heterogeneous [5]. Despite this high bat diversity, parasitological and molecular information on bat-associated filarioids in the Amazon remains limited, constraining current knowledge of their diversity, host associations, and geographic distribution [6]. Recent molecular studies in bats also emphasize unresolved taxonomic diversity and the value of integrative approaches for filarioid identification [7].

Among bat-associated filarioids, the genera *Litomosa* and *Litomosoides* (Onchocercidae) have been reported across multiple bat families, although their diversity, host specificity, and geographic distribution remain incompletely resolved [8]. Adult *Litomosoides* typically inhabit the thoracic and/or abdominal cavities of mammals, and infections in bats have been frequently documented in phyllostomids, including recurrent records in *Artibeus* spp [9]. *Litomosoides* infections are vector-borne, with dermanyssoid mites implicated in the transmission of some species. Recent reports of *Litomosoides* microfilariae in urban bats and their associated ectoparasites, including mites and bat flies, also support the inclusion of urban and peri-urban environments in surveys of bat-associated filarioid diversity [9, 10].

In Brazil, recent studies have increasingly combined conventional parasitological examination with molecular approaches to improve taxonomic inference and expand geographic and host records. For example, PCR and sequencing evidence extended the known distribution of *Litomosoides brasiliensis* to Maranhão and documented new host associations [6]. Nevertheless, large portions of the Amazon remain under-sampled for bat-associated filarioids.

Forest loss and fragmentation can modify bat–ectoparasite interactions [11], while urbanization can alter bat assemblage structure [12]. Because bat-associated filarioids have also been documented in urban environments [10], urban and peri-urban forest fragments represent relevant settings for surveys of parasite occurrence and diversity. Accordingly, the present study investigated the occurrence and molecular characterization of filarioid nematodes in bats captured in urban and peri-urban forest fragments in Rio Branco, Acre, Brazil, using complementary parasitological and molecular approaches.

## Materials and Methods

### Ethical Approvals and Permits

This study was conducted under authorization from the Brazilian Biodiversity Authorization and Information System (SISBIO; permit no. 83196-2) and was approved by the Animal Use Ethics Committee of the Federal University of Acre (CEUA/UFAC; protocol no. 31/2022). All capture and sampling procedures were performed by trained personnel and were designed to minimize handling time, stress, and the risk of injury.

### Study Area and Bat Sampling

**Fig. 1:**
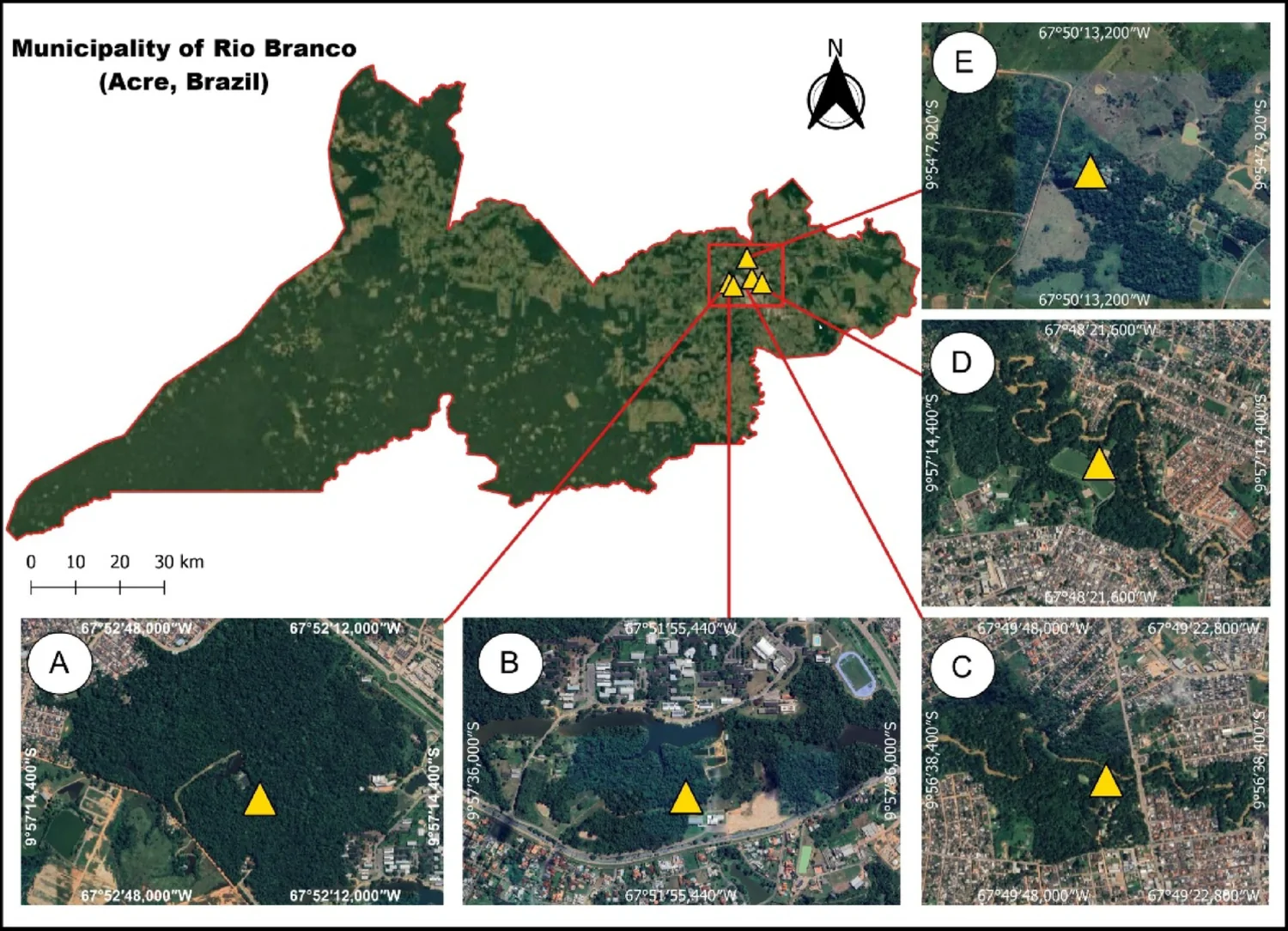
Bat capture sites in urban and peri-urban forest fragments in Rio Branco, Acre, Brazil (**A**–**E**)

Bats were captured in five forest fragments located in urban and peri-urban areas of Rio Branco, Acre, Brazil (Fig. 1), between December 2022 and October 2023. Sampling was conducted using seven mist nets (12 m × 2.5 m) deployed immediately after sunset and maintained open for 6 h per sampling night. Nets were inspected every 30 min to reduce stress, prevent prolonged entanglement, and ensure animal welfare. Net placement prioritized fragment edges and areas near water bodies, following standard bat survey procedures [13].

After capture, bats were carefully removed from the nets and temporarily housed in clean cotton bags for processing. Biometric measurements were recorded, and species identification was based on external morphology and morphometrics using taxonomic keys appropriate for Neotropical bats [14]. Each individual received a unique field code linking the capture site and date to laboratory sample identifiers.

### Blood Sampling

Prior to sample collection, bats were sedated by intramuscular administration of ketamine hydrochloride (Vetanarcol^®^, Laboratórios König, Brazil; 50 mg/mL) at a dose of 50 mg/kg [15]. After reaching an adequate anesthetic plane, animals were euthanized, and blood was subsequently collected by cardiac puncture using sterile 1 mL syringes fitted with 20 × 5.5 mm needles. When feasible, up to 1 mL of blood was obtained from each individual [16]. Whole blood was immediately transferred to tubes containing K₂-EDTA anticoagulant and gently inverted to prevent clot formation. Samples were then maintained under appropriate conditions until parasitological screening and DNA extraction.

### Parasitological Screening for Filarioid Microfilariae

Thin blood smears were prepared from EDTA-treated whole blood and stained using a rapid Romanowsky-type method (Panótico^®^). Slides were examined under light microscopy using 40× and 100× objectives, with fields scanned systematically for the presence of microfilariae [17].

In addition, the microhematocrit (Woo) method was used as a complementary screening technique [18]. Whole blood was loaded into capillary tubes, centrifuged, and the region adjacent to the buffy coat was examined under light microscopy for microfilariae. For each bat, both smear and microhematocrit results were recorded as positive or negative for circulating microfilariae.

### Molecular Detection of Filarioids and Sanger Sequencing

Genomic DNA was extracted from EDTA-treated whole blood using an in-house protocol adapted from Embrapa Gado de Corte for blood samples [19]. Briefly, 350 µL of blood was lysed with 20% SDS and incubated at 65 °C, followed by organic extraction with chloroform and a protein precipitation step. The aqueous phase was recovered, and DNA was precipitated with absolute ethanol (100%), washed with 70% ethanol, and dried to remove residual ethanol. DNA was then resuspended in 40 µL of nuclease-free water and stored at − 20 °C until PCR.

Filarioid DNA was detected by PCR amplification of an approximately 330-bp mitochondrial 12 S rRNA fragment using primers Fila12S-F (5′-CGGGAGTAAAGTTTTGTTTAAACCG-3′) and Fila12S-R (5′-CATTGACGGATGGTTTGTACCAC-3′), described by Otranto et al. [20]. PCR cycling conditions were as follows: initial denaturation at 95 °C for 3 min; 35 cycles of denaturation at 95 °C for 30 s, annealing at 60 °C for 30 s, and extension at 72 °C for 60 s; and a final extension at 72 °C. Amplicons were resolved by electrophoresis on 2% agarose gels. For each sample, 4 µL of PCR product was mixed with 1 µL of GelRed^®^ (Biotium, USA) and 1 µL of Blue Juice^®^ loading dye (Thermo Fisher) prior to loading. Bands were visualized under UV transillumination and recorded.

PCR-positive products were purified using ExoSAP-IT^®^ (Thermo Fisher) according to the manufacturer’s instructions. Purified amplicons were quantified by spectrophotometry and sequenced bidirectionally by Sanger chemistry using BigDye Terminator v3.0 on an ABI PRISM^®^ 377 genetic analyzer. Chromatograms were inspected, quality-trimmed, and assembled into consensus sequences using Geneious Prime^®^ 2022.1.1, generating the study contigs Contig-HF 17 and Contig-UDV 06 for downstream analyses. Contig-UDV 06 (324 bp; GenBank PZ124269) was obtained from *Artibeus planirostris*, whereas Contig-HF 17 (323 bp; GenBank PZ124268) was obtained from *Tonatia maresi*.

### Reference Dataset Assembly and BLASTn Taxonomic Assessment (12 S rRNA)

For comparative analyses, we compiled a mitochondrial 12 S rRNA dataset comprising the two sequences generated in this study—Contig-HF 17 (GenBank PZ124268; host: *Tonatia maresi*; deposited as Onchocercidae sp.) and Contig-UDV 06 (GenBank PZ124269; host: *Artibeus planirostris*; deposited as *Litomosoides* sp.)—and reference sequences representing *Litomosoides* spp. and closely related filarial taxa, including *Litomosa chiropterorum* and the outgroup *Acanthocheilonema reconditum* (total *n* = 18 sequences). Sequences were aligned in MEGA v12 using ClustalW with default nucleotide gap penalties, followed by manual inspection and trimming to remove poorly aligned terminal regions and standardize the alignment. After trimming, the final alignment used for downstream analyses comprised 300 nucleotide positions.

To provide an independent, database-driven taxonomic assessment of the study-derived sequences, each contig consensus sequence was queried against the NCBI nucleotide collection using BLASTn (database nt). Matches were evaluated based on percent identity, query coverage, and E-value, and the top-ranked hits were summarized to assess taxonomic affinity and contextualize divergence relative to available reference sequences.

### Pairwise Divergence, Similarity, and Phylogenetic Inference

Pairwise divergence was computed in MEGA v12 using p-distance, with substitutions including transitions and transversions, partial deletion, and a 90% site coverage cutoff. Under this criterion, sites with < 90% coverage were excluded from all pairwise comparisons, yielding a final dataset of 300 positions. Pairwise similarity was calculated as Similarity (%) = (1 − p-distance) × 100. The resulting similarity matrix was visualized as a heatmap in Python 3.13 using a custom script implemented in Spyder, with NumPy for matrix handling and Matplotlib for graphical rendering and export.

Phylogenetic relationships were inferred using maximum likelihood (ML) in MEGA v12 under the HKY + G model (Hasegawa–Kishino–Yano with gamma-distributed rate heterogeneity, using five discrete gamma categories). Tree searching employed the Nearest-Neighbor-Interchange (NNI) heuristic, starting from an automatically generated initial tree. Node support was assessed using 1,000 nonparametric bootstrap replicates, and the ML tree was rooted with *Acanthocheilonema reconditum*.

## Results

### Bat Captures and Taxonomic Composition

A total of 90 bats were captured across the five urban and peri-urban forest fragments (sites A–E). Captures were dominated by Phyllostomidae (81.11%, 73/90), followed by Vespertilionidae (10.00%, 9/90), Emballonuridae (6.67%, 6/90), and Noctilionidae (2.22%, 2/90). Overall, the bats represented 13 genera and 20 species. Species abundance varied markedly across the assemblage, with *Artibeus lituratus* accounting for the largest share of captures (27/90), followed by *Artibeus planirostris* (12/90) and *Carollia perspicillata* (10/90). The distribution of captures by species, family, and sampling site is detailed in Table 1.

**Table 1:** Distribution of bat species captured by family and sampling site (A–E) in Rio Branco, Acre, Brazil

Species	Family	A	B	C	D	E	Total
*Artibeus anderseni*	Phyllostomidae	–	–	–	–	2	2
*Artibeus cinereus*	Phyllostomidae	1	–	–	1	1	3
*Artibeus lituratus*	Phyllostomidae	5	5	6	9	2	27
*Artibeus obscurus*	Phyllostomidae	2	1	1	–	–	4
*Artibeus planirostris*	Phyllostomidae	2	–	5	1	4	12
*Carollia perspicillata*	Phyllostomidae	5	2	1	2	-	10
*Glossophaga soricina*	Phyllostomidae	–	–	–	3	-	3
*Hsunycteris thomasi*	Phyllostomidae	–	–	–	1	–	1
*Lophostoma silvicolum*	Phyllostomidae	–	–	2	–	–	2
*Macrophyllum macrophyllum*	Phyllostomidae	–	1	–	–	–	1
*Micronycteris sanborni*	Phyllostomidae	–	–	–	–	1	1
*Micronycteris hirsuta*	Phyllostomidae	–	–	–	1	–	1
*Myotis riparius*	Vespertilionidae	3	2	1	2	1	9
*Noctilio albiventris*	Noctilionidae	–	2	–	–	–	2
*Phyllostomus elongatus*	Phyllostomidae	1	–	–	–	–	1
*Phyllostomus hastatus*	Phyllostomidae	–	–	1	–	1	2
*Platyrrhinus incarum*	Phyllostomidae	–	–	–	–	1	1
*Platyrrhinus brachycephalus*	Phyllostomidae	–	–	1	–	–	1
*Rhynchonycteris naso*	Emballonuridae	–	–	–	–	6	6
*Tonatia maresi*	Phyllostomidae	–	–	1	–	–	1

### Parasitological Detection of Microfilariae

**Fig. 2:**
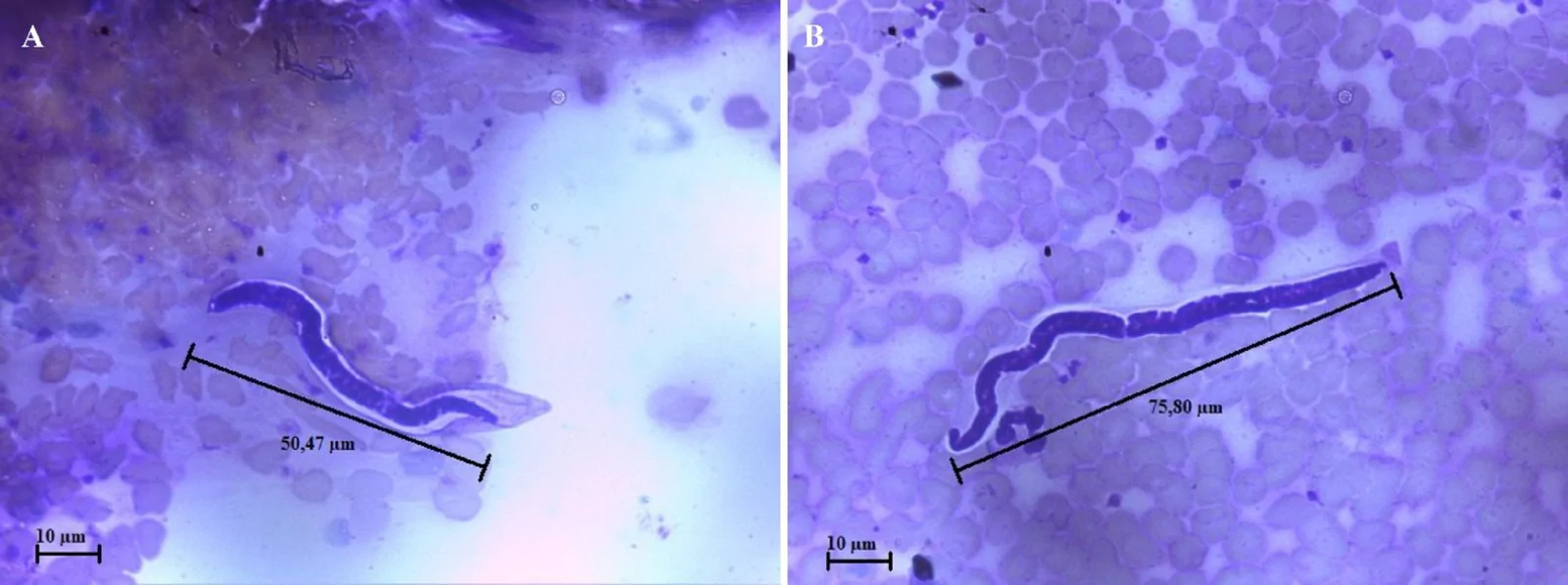
Microfilariae observed in blood smears from bats captured in forest fragments in Rio Branco, Acre, western Brazilian Amazon (1000×). **A** Microfilaria detected in a blood sample from *A. planirostris*, corresponding to Contig-UDV 06 (PZ124269). **B** Microfilaria detected in a blood sample from *T. maresi*, corresponding to Contig-HF 17 (PZ124268)

Direct parasitological screening by thin blood smear and microhematocrit (Woo test) detected circulating microfilariae (Fig. 2) in 2 of 90 bats (2.22%). Positive individuals were *Artibeus planirostris* (captured at site E) and *Tonatia maresi* (captured at site C). The positive *A. planirostris* yielded Contig-UDV 06 (PZ124269), whereas the positive *T. maresi* yielded Contig-HF 17 (PZ124268). Microfilariae were visualized under light microscopy at 1000× and were consistent with filarioid larvae based on their morphology and motility observed during screening.

### Phylogenetic Relationships, Pairwise Divergence, and BLASTn Taxonomic Assessment (12 S rRNA)

**Fig. 3:**
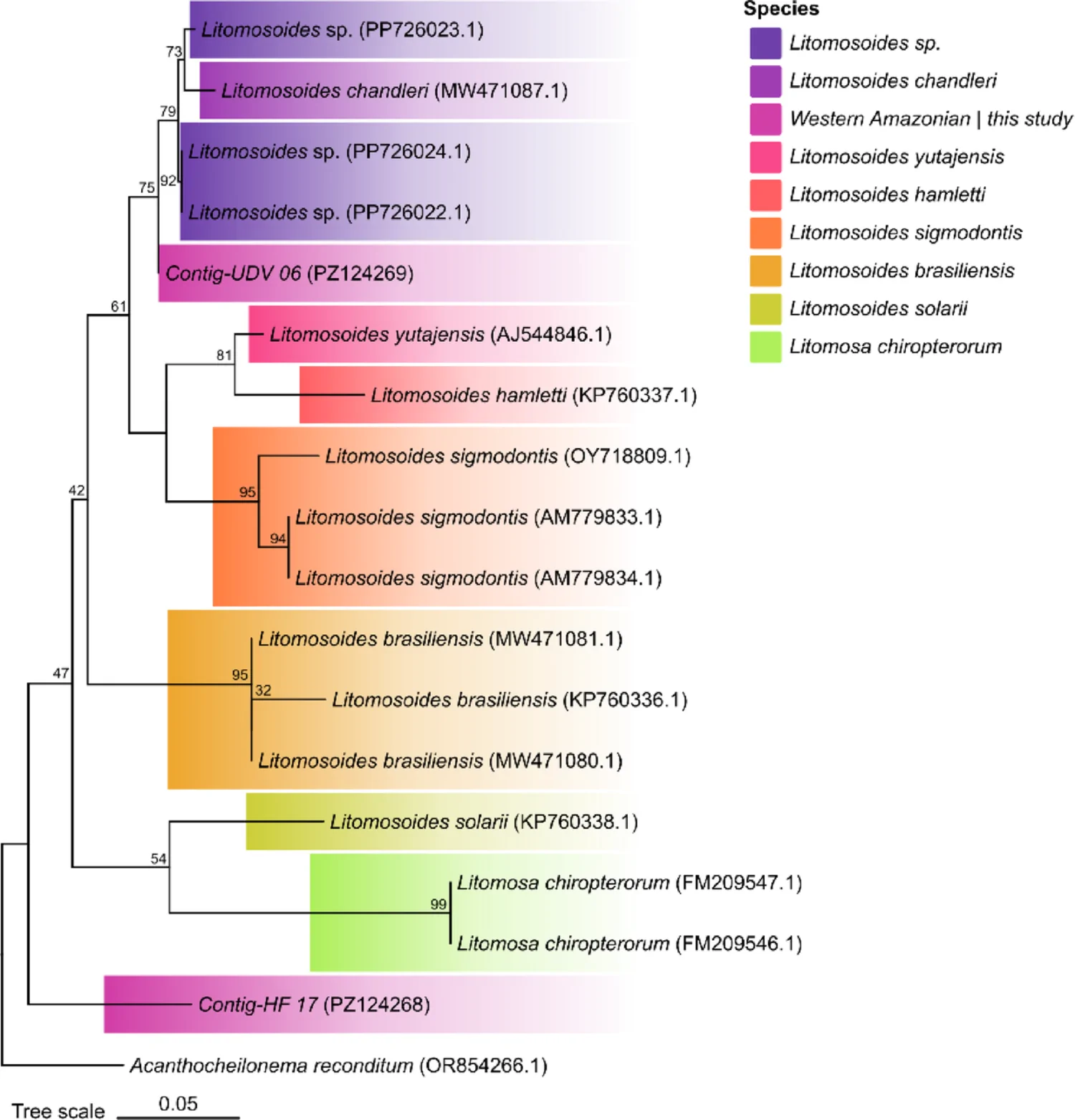
Maximum-likelihood phylogeny of filarial nematodes inferred from mitochondrial 12 S rRNA sequences under HKY + G and rooted with *Acanthocheilonema reconditum*, with node support assessed by 1,000 bootstrap replicates. Study-derived sequences are Contig-UDV 06 (PZ124269; host: *Artibeus planirostris*) and Contig-HF 17 (PZ124268; host: *Tonatia maresi*)

Maximum-likelihood (ML) analysis of the mitochondrial 12 S rRNA dataset (18 sequences; 300 sites after trimming and partial deletion at a 90% site coverage cutoff) placed the two study-derived contigs in markedly different phylogenetic positions (Fig. 3). Contig-UDV 06 (PZ124269), obtained from *Artibeus planirostris*, clustered within a *Litomosoides* group alongside *Litomosoides* sp. references and *Litomosoides chandleri*. Support across internal nodes within this cluster was moderate to high (bootstrap 73–92%), and the placement of Contig-UDV 06 relative to the *Litomosoides* sp. subgroup received additional support (75–79%), consistent with its close affinity to that lineage.

BLASTn results corroborated this inference: Contig-UDV 06 (PZ124269) returned top hits to *Litomosoides* sp. with approximately 98–99% identity and 100% query coverage, followed by *L. chandleri* with approximately 97–98% identity, all with highly significant E-values. Taken together, the concordant ML topology, full query coverage, near-complete identity, and low pairwise divergence support genus-level assignment of Contig-UDV 06 to *Litomosoides*.

Within *Litomosoides*, additional substructure was recovered, although support was heterogeneous. The *Litomosoides yutajensis* + *Litomosoides hamletti* grouping was supported (81%), whereas deeper separations among major *Litomosoides* lineages showed lower bootstrap values, approximately 40–61%, indicating limited resolution for older divergences at this fragment length. In contrast, within-species clustering was consistently well supported. The *Litomosoides sigmodontis* sequences formed a supported clade (95%), with strong support (94%) for the internal pairing of conspecific entries. The *Litomosoides brasiliensis* sequences also clustered together, with high support for the broader grouping (95%) but weaker support for internal branching (32%), again reflecting limited signal for fine-scale relationships within that taxon.

Outside the principal *Litomosoides* clusters, *Litomosa chiropterorum* formed a strongly supported pair (99%) and grouped with *Litomosoides solarii* with moderate support (54%). Contig-HF 17 (PZ124268), obtained from *Tonatia maresi*, was recovered as a divergent lineage outside the principal *Litomosoides* clusters, while its deeper placement within the ingroup was weakly resolved. BLASTn searches for Contig-HF 17 returned *Litomosoides* spp. among the closest available matches, but only at approximately 87–88% identity and 100% query coverage, together with similarly moderate identity values for other filarial taxa, such as *Dirofilaria* spp. at approximately 86%. Taken together, these results do not support confident assignment of HF17 to *Litomosoides*. Consistent with its GenBank annotation, HF17 is conservatively treated as Onchocercidae sp.

**Fig. 4:**
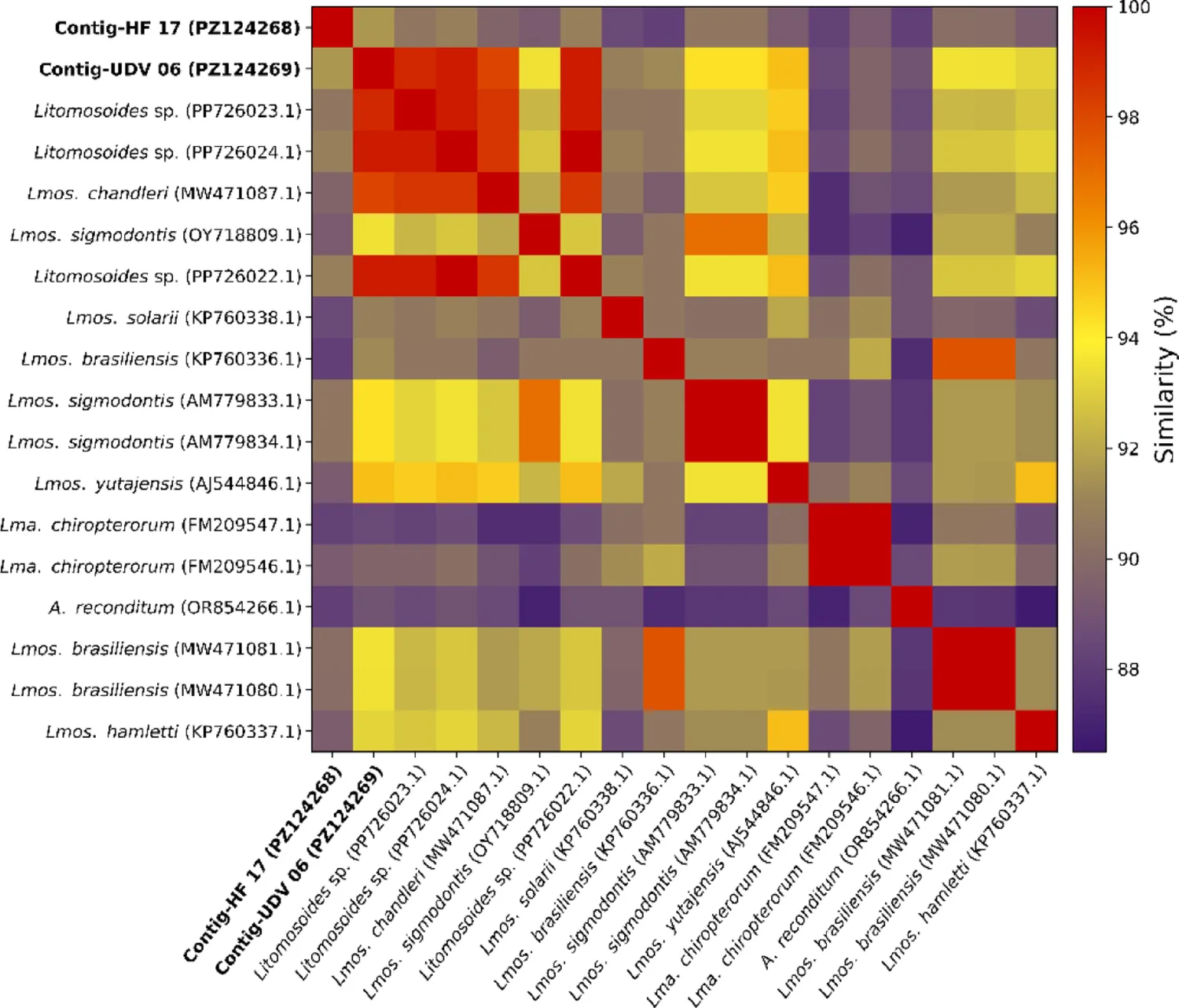
Heatmap of pairwise mitochondrial 12 S rRNA similarity (%) computed as Similarity (%) = (1 − p-distance) × 100 from the MEGA pairwise distance matrix using partial deletion, a 90% site coverage cutoff, and 300 retained sites. Labels show abbreviated taxon names and GenBank accession numbers. Study-derived sequences are Contig-HF 17 (PZ124268; host: *Tonatia maresi*) and Contig-UDV 06 (PZ124269; host: *Artibeus planirostris*), shown in bold. Abbreviations: *Lmos*., *Litomosoides*; *Lma*., *Litomosa*; *A*., *Acanthocheilonema*

Pairwise p-distance estimates were congruent with the main features of the ML tree (Fig. 4). Contig-UDV 06 (PZ124269) showed very low divergence from *Litomosoides* sp. references, consistent with its near-complete BLASTn identity. In contrast, Contig-HF 17 (PZ124268) was substantially more divergent from Contig-UDV 06 and from the *Litomosoides* sp. cluster. These distance patterns reinforce the strong *Litomosoides* affinity of UDV06 and the unresolved taxonomic placement of HF17. The similarity heatmap generated from the distance-derived similarity matrix summarized these relationships and highlighted the tight within-taxon similarity observed among conspecific references versus the broader divergence observed among genera.

## Discussion

This study combined parasitological screening and molecular analysis to characterize filarioid nematodes detected in two bat species from urban and peri-urban forest fragments in Rio Branco, Acre, western Brazilian Amazon. The two mitochondrial 12 S rRNA sequences showed markedly contrasting taxonomic patterns. Contig-UDV 06, obtained from *Artibeus planirostris*, showed strong molecular support for genus-level placement within *Litomosoides*. In contrast, Contig-HF 17, obtained from *Tonatia maresi*, was substantially more divergent and could not be confidently assigned to *Litomosoides* using the available short 12 S rRNA fragment. We therefore conservatively interpret HF17 as an unresolved member of Onchocercidae.

The taxonomic challenges encountered here reflect broader limitations in onchocercid systematics. A parasitic lifestyle has been associated with regression of morphological features and recurrent evolutionary convergence, complicating the reconstruction of robust phylogenetic hypotheses based exclusively on morphology [1]. Molecular approaches have substantially improved this scenario, particularly when integrated with traditional morphological and biological evidence [21, 22]. Early phylogenetic reconstructions based on ribosomal loci such as 5 S rDNA marked an important milestone in filarial systematics [23], and subsequent studies incorporated mitochondrial markers, including 12 S and *cox1*, as well as integrative DNA barcoding approaches for filarioid identification [22, 24–26]. Nevertheless, short single-locus fragments are often insufficient to resolve deeper relationships within Onchocercidae [1].

More recent molecular studies have revealed substantial evolutionary complexity within filarial nematodes, highlighting undescribed lineages and the need for continued taxonomic revision [1, 27, 28]. These findings reinforce the need for conservative interpretation of short single-locus sequences, particularly when available reference sampling is incomplete.

In our dataset, these limitations are most evident in the divergence profile of Contig-HF 17. Although BLASTn returned *Litomosoides* among the closest available matches, identity values of approximately 87–88% are too low to support confident assignment to that genus, and the sequence also showed moderate similarity to other filarial taxa. This pattern may reflect a divergent onchocercid lineage, incomplete representation of bat-associated onchocercids in public databases, or the limited discriminatory power of a short 12 S fragment for resolving deeper splits [29, 30]. Its deeper phylogenetic position should therefore be interpreted cautiously, because weakly resolved nodes, taxon sampling, and long-branch effects can influence phylogenetic placement [31, 32].

These gaps are especially pronounced in the Amazon, where bat-associated filarioids remain under-sampled from both parasitological and molecular perspectives, despite recent records expanding the known distribution and host associations of *Litomosoides* in the Legal Amazon [6]. Our findings add two host-associated molecular records from the western Brazilian Amazon: UDV06 is closely related to known *Litomosoides* sequences, whereas HF17 represents unresolved onchocercid diversity associated with *Tonatia maresi*. Given that only two bats were positive, these findings should be interpreted primarily as evidence of parasite occurrence and molecular diversity rather than as estimates of transmission patterns.

Although Onchocercidae includes taxa of recognized medical and veterinary importance [33, 34], no zoonotic relevance can be inferred from the filarioids detected here. Urbanization can alter bat habitat use and community composition [35–37], while forest loss and fragmentation can modify bat–ectoparasite interactions [11]. However, the present study did not sample ectoparasites or other potential vectors. Future surveys incorporating bat-associated ectoparasites could test vector associations and transmission hypotheses [10, 38, 39]; however, the present data do not permit conclusions about local transmission pathways.

The contrast between UDV06 and HF17 defines a focused taxonomic question for future investigation. UDV06 shows strong molecular support for placement within *Litomosoides*, whereas HF17 remains unresolved within Onchocercidae. Multilocus data, particularly mitochondrial *cox1* together with an independent nuclear locus and expanded reference sampling, are required to determine whether HF17 belongs to *Litomosoides* or to another onchocercid lineage [29, 40].

The present analysis is also limited by the short 12 S rRNA fragment, with 300 aligned positions retained for phylogenetic comparison. Longer mitochondrial regions and complete mitogenomes can provide additional information for filarial systematics and genetic differentiation [41, 42]. Clear reporting of alignment and trimming decisions remains important because these choices can affect distance estimates and node support [43, 44]. When deeper nodes receive weak support, interpretation should emphasize well-supported terminal groupings together with complementary evidence from BLASTn identity, query coverage, and pairwise distances [32].

Overall, our findings expand current knowledge of bat-associated filarioid diversity in the western Brazilian Amazon. Future work should prioritize broader bat sampling, expansion of reference sequence databases, and multilocus molecular characterization using mitochondrial and nuclear markers. Vector sampling may be incorporated into dedicated studies of transmission, but the present results support conclusions primarily on parasite occurrence, host association, and taxonomic diversity [1, 22, 28, 29].

In summary, circulating filarioid microfilariae were detected in *Artibeus planirostris* and *Tonatia maresi* from the western Brazilian Amazon. The 12 S rRNA sequence obtained from *A. planirostris* (UDV06) showed strong molecular support for genus-level assignment to *Litomosoides*, whereas the sequence obtained from *T. maresi* (HF17) was substantially more divergent and could only be conservatively assigned to Onchocercidae. These findings provide new molecular evidence of bat-associated filarioid occurrence and diversity in the region while highlighting the limitations of short single-locus sequences for resolving divergent onchocercid lineages. Additional mitochondrial and nuclear markers are required to clarify the taxonomic position of HF17.

## Data Availability

The sequences generated in this study are publicly available in GenBank under accession numbers PZ124268 (Contig-HF 17) and PZ124269 (Contig-UDV 06). Additional data supporting the findings of this study are available from the corresponding author upon reasonable request.

## Code Availability

The custom Python script used to calculate the similarity matrix from the MEGA p-distance output and generate the heatmap is available from the corresponding author upon reasonable request. All other analyses were performed using publicly available software, as described in the Materials and Methods section.
